# Phonological Awareness as the Foundation of Reading Acquisition in Students Reading in Transparent Orthography

**DOI:** 10.3390/ijerph18105440

**Published:** 2021-05-19

**Authors:** Vesela Milankov, Slavica Golubović, Tatjana Krstić, Špela Golubović

**Affiliations:** 1 Department of Special Education and Rehabilitation, Faculty of Medicine, University of Novi Sad, 21000 Novi Sad, Serbia; spela.golubovic@mf.uns.ac.rs; 2 Faculty of Special Education and Rehabilitation, University of Belgrade, 11000 Belgrade, Serbia; slavica.golubovic@yahoo.com; 3 Department of Psychology, Faculty of Medicine, University of Novi Sad, 21000 Novi Sad, Serbia; tatjana.krstic@mf.uns.ac.rs

**Keywords:** orthographic transparency, phonological awareness, reading, reading difficulties

## Abstract

Phonological skills have been found to be strongly related to early reading and writing development. Accordingly, the aim of this study was to examine the extent to which the development of phonological awareness facilitates reading acquisition in students learning to read a transparent orthography. Our research included 689 primary school students in first through third grade (Mean age 101.59 months, SD = 12,690). The assessment tools used to conduct this research include the Phonological Awareness Test and the Gray Oral Reading Test. According to the results from the present study, 13.7% of students have reading difficulties. Students with reading difficulties obtained low scores in phonological awareness within each subscale compared to students who do not have reading difficulties (*p* < 0.01). Components of phonological awareness which did not singled out as strongly related to early reading success include Phoneme Segmentation, Initial Phoneme Identification, and Syllable Merging. Thus, understanding the nature of the relationship between phonological awareness and reading should help effective program design that will be aimed at eliminating delayed development in children’s phonological awareness while they are still in preschool.

## 1. Introduction

During their development, a large number of children do not experience difficulties in learning early literacy skills. However, a number of children struggle with reading challenges over a long period of time, whereas some of them do not even reach the expected level of these skills [1]. Orthographic transparency is an important factor influencing reading acquisition and refers to the relationship between written symbols—graphemes representing speech sounds—and phonemes. The research on alphabet writing systems indicates that in language, orthographic transparency is increasingly recognized as important when determining the degree of difficulties with learning to read. A transparent orthography has a simple one-to-one relationship while less transparent orthographies are those in which the relationship is more complex.

Orthographic decoding is not equal in all languages. Arabic and Hebrew writing systems, for example, require more developed visuospatial abilities and better visual attention skills, necessary for decoding, compared to those in English [2,3,4,5,6].

According to the orthographic depth hypothesis, shallow orthographies are more easily able to support a word recognition process that includes phonology, and children learn to read relatively quickly. Phonological awareness and the ability to decode words into their constituent sounds are cited as predictors of literacy. In contrast, deep orthographies encourage a reader to process printed words by referring to their morphology through the visual-spelling structure of the printed word, thus having much more difficulty in decoding words. As a result, reading is slower to learn [7].

The tradition of literacy in the Republic of Serbia is neither as long nor as rich as, for example, in England, Germany, France or Russia, but the orthography of the Serbian language is simple and transparent and should not create problems in mastering it. Nevertheless, a large number of completely or functionally illiterate individuals or social groups are registered in Serbia. What is worrying is the large percentage of children who remain functionally illiterate [8].

Over the last thirty years, particular attention has been paid to phonological and phonemic awareness that lays the foundation for success in acquiring and developing reading skills [9,10,11,12,13,14,15].

Phonological awareness involves the auditory and oral manipulation of sounds, whereas phonemic awareness refers to the ability to understand the relationship between the written symbols—letters that represent the sounds in spoken words—and it could be therefore said that a phonological awareness is a broader notion than phonemic awareness [16,17,18].

Phonological awareness is the ability to identify, process, and manipulate phonological units that compose spoken words of different complexity and size [19]. It includes the understanding of different ways that the words in our spoken language can be broken down into their various components which can be then manipulated [1].

Phonological awareness in children, especially in the early stages of reading, improves and accelerates learning to read, and at the age of six it is a strong predictor of their future reading ability. Hence, a child’s level of phonological awareness acquisition accounts for the child’s readiness to read. Equally, phonemic awareness has been singled out as a crucial factor when it comes to reading nonsense words and text comprehension [20,21].

In addition, syntactic awareness has been found to be an important support for phonological awareness and a significant predictor of reading accuracy. This is especially important for children who read more complex texts and have likewise overcome reading and decoding individual words. Thus, phonological skills are important predictors of the initial reading and writing development [11,15,16]. Deficit in ability to understand the phonological structure of language has been found to be the primary cause of developing later reading difficulties [10,12,13,14,22]. Thus, phonological awareness has been regarded as the major and most widely accepted cause of dyslexia so far [23].

Phonological deficit explains reading difficulties as a consequence of impaired phonological processing skills which manifests itself as a deficit in the ability to understand the relationship between graphemes and phonemes [24,25].

Consequently, children with reading difficulties have been shown to have impaired phonological awareness, but may also have impaired verbal short-term memory, rapid automatized naming, speech perception, and perception of visual forms [26,27,28,29]. Deficits in phonological awareness and automatic rapid naming skills are particularly pronounced among individuals with dyslexia [30].

In other words, they performed less-well on tasks that are fundamentally dependent on distribution of phonological representations which affect accessibility and revocation of phonological information: phonological processing capabilities resulting from ill-formed phonological perceptions [15,31,32].

Although the relation between phonological skills and reading rely on the assumption that it is bidirectional, the nature of these skills and their precise effects on the development of reading has been a matter of a constant controversy [22]. Thus, the aim of this study was to investigate the extent to which the development of phonological awareness contributes to reading acquisition in students reading in transparent orthography. Additionally, our aim was to adapt the reading test in relation to linguistic and cultural differences, while retaining metric characteristics such as reliability and validity. We expected that children who have a problem with phonological awareness to have more difficulty reading.

## 2. Materials and Methods

### 2.1. Participants

The research comprises primary school students from first through third grade from three towns. The towns, which have approximately the same number of inhabitants, are located in the northern part of the Republic of Serbia. All schools are state-owned institutions with the same educational system. Prior to conducting this research, ethical approval was obtained from Ethics Committee of the Medical Faculty, The University of Novi Sad (Decision No. 52/14), and then the written permission from school principals in which the research was conducted, as well as the written consent from parents of students involved in the research.

First, invitations to participate in this study were sent to all parents (a total of 1136), of which 946 (83.27%) gave their written consent to participate. Subsequently, students engaged in inclusive education were excluded from the sample, as well as students with uncorrected sensory impairments (hearing and vision), students with intellectual impairment, neurological and psychiatric disorders or severe motor impairments, students with speech and language disorders and students whose first language is not Serbian. The research began with a total of 810 (85.62%) students, but complete responses were obtained from 689 (85.06%) students, which is the final number of students included in this study.

The research comprised 116 first grade girls and 128 boys, 94 s grade girls and 116 boys, while 109 girls and 126 boys were from the third grade (Table 1). There is a gender balance among the groups of the first, second and third-grade students (χ2 = 0.35; *p* = 0.83).

Data, when initially obtained, were analyzed using χ2-test, and the results of the study indicate that there is no significant difference between students of both genders in any of the three groups of students formed based on the grade level (χ2 = 0.35; *p* = 0.83). In order to exclude students whose intellectual abilities are below average from the sample we used data from school protocols obtained by Raven’s testing progressive matrices [33] for participants enrolled in Grades 1, 2 and 3. All students were tested before starting school. The use of this data was approved by the school principal, as well as the students’ parents who confirmed this by giving their written consent. Available data on students were whether their achievement on the test was below average, average or above average. The study inclusion criteria involved students with an IQ of 90 and >90. Children who had below-average achievement when enrolling in school did not take this test included in the study.

All students come from urban areas and both students and their parents are native Serbian speakers. Regarding the parental educational levels, the highest percentage of parents had secondary level of education (63%), while 29% had higher level of education and 8% had primary school education.

### 2.2. Instruments

The Phonological Awareness Test—FONT—was used to measure phonological awareness [19]. The test contains six items, within each are eight types of tasks, for which permission has been obtained from the author. The tasks included: syllable merging, syllable segmentation, initial phoneme identification, rhyme recognition, phoneme segmentation, final phoneme identification, phoneme deletion and phoneme substitution. The test was constructed and validated for the Bosniak and Serbian languages, and it was therefore applicable for assessing students who participated in our research. According to the authors of the test, the specified time required to assign tasks is limited, but in their pilot study the scheduled time proposed to assign all eight tasks, with six items each, was about 30–45 min.

In our study, the scheduled time required to assign the tasks was about 25–35 min. The responses are assessed at each age level. The categorization with the respect to the achieved success in the test is the following: Below Average, Low Average, Average, High Average and Above Average. Accordingly, students were classified into these categories according to their test achievements.

In testing, all tasks have been solely formatted to assess tasks of oral production. In view of this, they were chosen to represent the most typical and appropriate forms of words in Serbian language. The test does not include tasks that denote non-words in Serbian, such as items that require phoneme manipulation in the middle of the word, or could lead to a change in the type of word, noun gender, or the case. Each task contains six items that are scored based on false-true response options. In the test, items (except for control items) were ranked according to the item difficulty. According to the author of the test, a very high test reliability was determined (Cronbach’s ά = 0.96) after standardization, with approximately normal distribution of corrected item-total correlations with acceptable range and average value, which indicates good internal consistency and item discrimination within the included age interval. First, for the purpose of the present research we conducted a pilot study to validate the test on the sample of 46 first grade primary school students (23 boys and 23 girls), average age 7.08 years, standard deviation (SD) 0.50). The results of the pilot study showed high reliability of the test (Cronbach’s ά = 0.95), as well as that there are no differences in relation to gender and phonological awareness, and subsequently the instrument was applied to the entire sample. The validation has been performed since the Ekavian subdialect is used in the Republic of Serbia, while the authors of the test use the Ijekavian subdialect represented in Bosnia and Herzegovina. The difference between the two sub-dialects could affect the understanding of the words presented in the test.

The results obtained on the entire sample show high reliability for both the instrument as a whole and for the subscales (Table 2).

Verification of the assumption for the dimensionality of the questionnaire was performed by exploratory factor analysis, the method of principal axes (Principal axis factoring) in SPSS 22 (IBM Corp., Armonk, NY, USA). Significant Bartlett’s test of sphericity [χ2 (1128) = 14,141,492; *p* < 0.001] indicate that the inter-correlation matrix is factorial. The Unweighted Least Squares (ULS) procedure was used to extract the number of factors. In order to determine the number of significant factors, the data were subjected to bootstrap parallel analysis (1000), integrated into the FACTOR program (Lorenzo-Seva & Ferrando, 2006, Tarragona, Spain, available: https://psico.fcep.urv.cat/utilitats/factor/index.html). It was found that the four characteristic roots of the actual data explain the higher percentage of variance than their random counterparts according to the criterion’s 95th percentile, and the authors opted for that number of factors, since it also met the criterion of interpretability. These four factors explain 52.321% of the variance. Promax rotation was used, and the correlation matrix is shown in Table 2. Only loads exceeding the value of 0.30 are shown in the table. Based on the correlation matrix, it can be noticed that no item has cross-loads on more than one factor, while the 3 items of the Initial Phoneme Identification have loads less than recommended, in order to be kept in the scale. The first factor is called Stylistic Awareness and encompasses all items of the Syllable Merging and Syllable Segmentation subscales. Apart from the items from these two subscales, this factor does not contain saturations of other items from other subscales and explains 28.99% of the variance. The second factor is termed Advanced Phonemic Awareness and encompasses all items on the Phoneme Elimination and Phoneme Substitution subscales and explains 10.128% of the variance. The third factor, called Initial Phoneme Awareness, is mostly saturated with items from the Rhyme Recognition subscale, although it also contains significant factor loads on 3 of the 6 items of Identification of the Initial Phoneme. The remaining three items of this scale do not achieve significant saturation on any factor. The third factor explains 7.429% of the variance. The fourth factor is saturated only with the items of the Phoneme Segmentation subscale, and it is named the same as the mentioned subscale and explains 5.765% of the variance. This factor includes 5 of the 6 saturated items on this subscale, while the remaining are achieved in the form of low saturated first factor. The reliability of individual factors has been found to be good, ranging from 0.83 for the Initial Phoneme Awareness factor, and 0.84 for the Phoneme Segmentation factor; to 0.87 for the Advanced Phoneme Awareness factor and 0.92 for the Syllable Awareness factor. Considering the whole of the scale the reliability is very high amounting to 0.95. The correlations between the factors are low and range from 0.133 to 0.420, and therefore there was no need to check the second-order factor structure. Factor scores were preserved as variables and thus formed scores of four subscales which were used in further analyses.

Furthermore the Gray Oral Reading Test (GORT 5) is one of the most commonly used measures of oral reading fluency and comprehension [34]. It is designed for identification of dyslexia in students ranging in age from 6 years to 23 years and 11 months, as well as for students who may need more intensive and explicit instruction in reading acquisition. According to the authors of the test, reliability Chronbach alpha was 0.90. The test includes tasks that assess oral reading speed, accuracy, fluency and comprehension on the basis of chronological age and grade. The oral reading index is a composite score formed by combining students’ fluency and reading comprehension scores [35].

In the Republic of Serbia, there is no standardized reading assessment tool that could be used with the general population for measuring essential components of reading, such as speed, accuracy, fluency and reading comprehension. Taking into account that the original version of the test is in English, the process of a cross-cultural adaptation of the test was conducted to best reflect the aims of this research, following the guidelines for adaptation with regard to linguistic and cultural differences [36]. According to translation and adaptation guidelines, the forward translation from English to Serbian was done first, independently by two translators. One of the translators was, by recommendation, of the same occupation, and the other had no experience related to this field. Then, a synthesized version was produced and two translations were harmonized into one version by two translators. Next, it was translated from Serbian into English, undergoing the process of the so-called double blind translation, followed by harmonization of translators and examiners in the field of semantic equivalence, idiom equivalence, experiential and conceptual equivalence of concepts. There was no need for changes in the content. Following this phase, we moved through the pilot study to implement this test on a sample of forty third-graders and the same number of second-graders. The obtained results showed high reliability (Cronbach’s ά = 0.91) and the instruments were applied to the entire sample.

The results found in the entire sample show high instrument reliability in relation to the Comprehension and Oral Reading Index (Table 3). Since the other GORT test assessment measures showed low reliability, in view of the fact that the Oral Reading Index is a composite score formed by combining fluency and comprehension scaled scores, in further analysis we will use only Total Reading Index since the Oral Reading Index represents the most reliable testing score. Indices in the group of average to high (over 90) are achieved by students who have reached the level of oral reading that is expected for their age. Low indexes (below 90) are given to students who have not reached the level expected for their age in reading.

### 2.3. Study Design

Before starting the test, parents were informed about the purpose and procedures associated with the assessment. First, they were given oral explanation, and then printed information. For parents who were not present, written information and an envelope were provided to send/return their completed consent to the examiner. Students would participate in the survey provided that their parents had given consent for their child’s participation in the research.

The assessment took place in the classrooms that students regularly attended. Classrooms were isolated from noise and distracting sounds. The assessment was performed both during students’ regular and extended stay in school. As a rule, students entered the classroom one at a time and first got to know the examiner via informal conversation. First grade students were included in the research in the second term, since the education system in Serbia is organized so that children in the first term of the first grade master the basics of reading and only in the second term are they expected to read independently.

Students were first assessed using the FONT test. It was explained to them that they should answer the examiner’s questions with YES or NO, and that the questions would be asked orally. At all times, they had the opportunity to seek further clarification from the examiner.

Subsequently, reading performance was tested. Students were first given explanation about reading the text, and answering a few questions about the texts read. They were told that the examiner would record some data while they were reading, and that it was non-evaluative assessment.

### 2.4. Statistical Analysis

Data analysis was performed using the statistical package SPSS 20 (IBM corp., Armonk, NY, USA).

Descriptive statistics methods were used to measure central tendency (arithmetic mean), and measures of variability (standard deviation) in order to summarize the major numerical characteristics of observations. Additionally, we applied factor analysis, one-factor ANOVA, MANOVA, T-test and Regression Analysis. In the tests used, statistically significant differences were observed outside the 95% confidence interval (*p* < 0.05). To measure the reliability across the whole scale, the Cronbach’s alpha coefficient was used as a measure of internal consistency. The coefficients of at least 0.80 were considered acceptable.

## 3. Results

Reading success was first analyzed on the entire sample. The obtained results show that students classified to the category of Very Poor made up 2.3%; Poor 11.2%; and Below Average 32.6%; whereas the largest number of students from first through third grade were classified to category 4, which corresponds to the category of Average (47.8%); Above Average made up 3.2%; Superior 2.0%; and Very Superior reading 0.9%. There is no significant difference in relation to the frequency-based categories of students’ reading success in relation to the class they attend (χ2 = 9.97, *p* = 0.61).

Additionally, one-way analysis of variance was used to test whether there are differences in students’ scores in different classes in relation to the Oral Reading Index scale. The results show that there is no significant difference in the overall index of oral reading (F = 0.45, *p* = 0.64) in relation to the class that students attend (Table 4).

In relation to the level of development of Phonological Awareness, five categories have been formed. In relation to the class that the child attends, differences were noticed in relation to the category of development of Phonological Awareness (χ2 = 378.71, *p* < 0.001). Table 5 shows that in the first grade there are significantly more students in the Lower Average category compared to the number of students in this category of Phonological Awareness in the second and third grade. Additionally, in the second grade there are significantly more students who are in the category Below Average compared to the third grade students. In the second and third grade, there are significantly more students in the Above the Average category as compared to the first grade. According to the instructions given in the FONT Assessment Manual, there is no Above Average category for the second and third grade and for that reason a 0 value is given for these columns.

In our further analysis we were interested in if there is a significant difference in the level of development of certain elements of phonological awareness among students of different grades (Table 6). This question was checked by MANOVA in order to prevent the occurrence of an alpha error. Box’s Test of Equality of Covariance Matrices shows that there are no differences in the covariance of the dependent variables between the groups. Pillai’s Trace MANOVA test shows that four subscales of the FONT test show differences in relation to the grade that students attend (F = 1,790, *p* < 0.001). The magnitude of the effect shows that 8.5% of the differences between classes are explained by four subscales. In the next step, individual ANOVAs were performed. In relation to all four subscales/factors of the FONT scale, there is a significant difference in relation to which grade the student attends.

On all the Phonological Awareness subscales, it was noticed that there is a significant difference in the manifestation of scores among students who attend different grades of primary school. Syllable Awareness, Advanced Phoneme Awareness and Phoneme Segmentation are significantly more developed in the third grade students compared to all younger students, while the second graders have a significantly better result than the first-graders. On the Initial Phoneme Awareness subscale, the second-graders are significantly better than the first-graders., but no differences were observed between second and third grade students. However, the magnitudes of the effects of these differences are small, less than 0.2.

In order to examine the differences in the phonological awareness of children with and without reading difficulties, we formed groups based on the Oral Reading Index, so that the children with an Oral Reading Index below 90 entered the group of children with reading difficulties. The MANOVA results show that the students with reading difficulties have poorer overall phonological awareness than students who do not have reading difficulties. The overall model is significant (F = 12.908, *p* < 0.001, d = 0.070). When the differences in relation to each subscale of phonological awareness were analyzed, it was noticed that differences are observed in the same direction on the first two subscales, while there are no significant differences on the other two subscales (Table 7). In terms of measures of magnitude, these differences were expressed using the Cohen’s *d* coefficient, which ranged from d = 0.014 to d = 0.053. The magnitude of the effect shows that these significant differences are also very small (Table 7).

Although the correlations between the overall Reading Index and the FONT total (r = 0.19, *p* < 0.001) and subscales are significant (ranging from 0.07 to 0.20), in order to examine how much of the variance in the oral reading index can be explained based on four phonological awareness variables, multiple regression analysis was applied (Table 8). The set of predictors consisted of Syllable Awareness, Advanced Phoneme awareness, Initial Phoneme awareness and Phoneme Segmentation. Preliminary analyses were conducted to determine that there were no significant deviations from the expected normality, linearity, multicollinearity, and homoscedascity.

The obtained model is statistically significant, F (4,688) = 13,356, *p* < 0.001. The multiple co-relation coefficient is R = 0.269. The percentage of variance of the overall Reading Index explaining predictor variable is 7%.

A significant positive contribution of the two separate predictor variables was observed, whereby the initial phonological awareness scale has a higher beta coefficient (Beta = 0.212, *p* < 0.001) than advanced phoneme awareness scale (Beta = 0.096, *p* = 0.046) (Table 9).

## 4. Discussion

Contemporary scientific considerations support the phonological awareness deficit theory as the leading cause of dyslexia and reading difficulties. Considering that the Serbian language belongs to the group of transparent languages, in which one grapheme corresponds to one phoneme, this letter-to-sound conversion method should be facilitating literacy acquisition and learning to read. Thus, students who are learning to read consistent or transparent orthography, with letter-sound correspondence, learn to read faster [37].

Additionally, the results obtained show that a large number of children included in this study have reading problems, which is a key criterion for classification to the category of reading difficulties. In our research, below low and low reading skills were found in 13.5% of students, while the largest number of students from first through third grade were average readers. The previous research in our country, which referred to identifying students with reading difficulties, was conducted in 1999 and then 8.4% of children with reading disabilities were reported [38]. In recent years, we have seen an increase in the number of students with reading difficulties, which can be justified by the greater sensitivity of the academic community to this problem, in addition to better recognition and greater involvement of experts in this field. Another reason for the increase in the number of students with reading difficulties is that previously there was no instrument to measure characteristics that would clearly define the difference between average reading and reading difficulties, and the grading was based on the examiner’s subjective evaluation. By using GORT 5 and FONT, we have tried to point out the importance of applying valid instruments for reading assessment and readiness to read.

Children with reading difficulties, compared to typical readers of their same age, have substantially lower achievements in reading accuracy and speed [39]. Further, the results of the present study show that reading accuracy was a minor problem with regard to reading speed, while most common error types were sound and syllable omissions or addition in words. In the research that comprised native German speaking children with reading difficulties [40], as well as in our research, reading speed deficits for all types of reading tasks, including text were found. Additionally, error types correspond to those of native Serbian speaking children. Accordingly, the results of this research have shown that students who are slower in reading, in terms of accuracy, have more pronounced problems related to automatization of components of phonological awareness because it has not evolved over time. These results are in accordance with other studies confirming that students with reading difficulties are found to have reading speed as a core deficit because phonological awareness skills have not been automated [39]. Furthermore, the naming speed is related to the reading speed and the number of incorrectly read words errors [41]. These findings offer support for the hypothesis of reading speed as the core reading deficit in a transparent orthography.

Reading difficulty is associated with phonological awareness and the ability to identify words. The school system in Serbia prescribes children to enroll in the first grade at the age of seven, so that the students included in our sample were older than the students in similar research, in which the abilities of phonological awareness were assessed. Thus, the successful performance of students in our sample can be explained, with more than half of students who achieved above average phonological awareness scores. The results of this research show that students who have been classified as those with low phonological awareness have less developed vocabulary compared to students with average and above average phonological awareness measures. In the second grade students, low average phonemic awareness scores also correlates with lower vocabulary subtest scores. Actual result was expected for second grade students, because poor vocabulary and limited lexical resources can impoverish their knowledge of phoneme-grapheme relationship in words, i.e., phonological awareness. Studies conducted with native English speaking children had also reported very good children’s phonological awareness at the age of four and five. For them, performing tasks involving rhyme recognition were easier than recognizing the initial phoneme. Students who had been learning how to read for about a year were able to perform syllable and phoneme segmentation [42,43].

Goswami’s research [44] suggests that a sequence of a phonological awareness development is universal across languages. Evidence of this lies in phonological skills such as syllable segmentation, initial phoneme identification, and rhyme recognition, which begin before developing literacy skills [44,45]. Cassady et al. [46], as well as Rathvon [47] suggest that all types of phonological awareness tasks can be grouped into nonphonemic tasks (which measure global aspects of phonological awareness, such as rhyming and syllable segmentation) and phonemic awareness tasks (which measure the ability to attend to or to manipulate individual phonemes).

By analyzing separate components of phonological awareness compared to chronological age, it was noticed that the first grade students in our research were worse at performing tasks of syllable merging, syllable segmentation, identifying the initial phoneme and recognizing rhyme compared to second and third grade students. Students who have better developed phonological skills are better at reading fluency, as well as reading comprehension. Components of phonological awareness that are formed before entering school and acquisition of literacy skills are not closely connected to speed, accuracy, fluency and reading comprehension. This can be explained by the fact that syllable awareness, sound segmenting and initial phonological awareness, elements of phonological awareness, are important for the pre-reading period. Already at the end of the first, and especially the second grade, the tasks with the elimination of the initial phoneme, the identification of the final phoneme and phoneme substitution, i.e., advanced phonological awareness, proved to be more difficult. This can be attributed to the fact that syllable and phoneme segmentation, initial phoneme identification and rhyming are components of phonological awareness that are important for the pre-reading learning period. Until the end of the first, and particularly second grade, the tasks in initial phoneme deletion, final phoneme identification and phoneme substitution turned out to be more difficult. Students in the second and third grade were more successful in performing these tasks compared to the first grade students, which suggests contribution of reading process to the improvement of phonological awareness.

At the same time, research shows that already preschool—aged children can successfully segment words into syllables, while phoneme segmentation is successfully performed only with starting school and learning to read [45,48,49,50,51,52]. Therefore, the data obtained from this study confirm the findings of previous studies. Likewise, first grade students have shown more developed syllable segmentation compared to phoneme segmentation, whereas the other components of phonological awareness were developed in the second and third grade students. We assume that this happened as a result of reading and literacy acquisition.

The primary deficit that underpins reading difficulties in all languages lies in problems with phonological awareness [44,53,54]. In this context, it is recommended to focus on five cognitive strategy and skill areas that contribute to reading development—phonological awareness, learning of grapheme-phoneme correspondences, reading fluency, vocabulary and reading comprehension [55,56,57]. There was the same tendency in the present research. The group of students whose reading scores did not involve deviation below the average had shown a more developed phonological awareness and were more successful in all reading tasks. Students who had lower reading scores also obtained lower scores in the phonological awareness test.

## 5. Conclusions

Our research showed that the largest number of students who participated in the research had an average reading development. There was no significant difference in reading among students of different grades. Students who have difficulty reading have poorer phonological awareness compared to students who have no difficulty reading. Phonological awareness has proven to be a significant predictor in mastering reading on two subscales, but even these significant differences are very small.

The limitation of this study lies in the fact that an additional instrument wasn`t used for the assessment of reading in order to establish concurrent validity of the GORT 5 test, which was used for the first time in Serbian. The shortcoming of our research is not taking into account the time devoted to reading instruction and reading preparations done at pre-school institutions. Subsequent studies should also consider the time that the children spend reading at home, as well as different reading instruction styles.

## Figures and Tables

**Table 1:** Sample of participants.

	All	1st Grade	2nd Grade	3rd Grade
Boys	370	128	116	126
Girls	319	116	94	109
	689	244	210	235

**Table 2:** Correlation matrices.

	Factor I	Factor II	Factor III	Factor IV
Syllable merging 1st item	1.00			
Syllable merging 2nd item	0.719			
Syllable merging 3rd item	0.617			
Syllable merging 4th item	0.716			
Syllable merging 5th item	0.601			
Syllable merging 6th item	0.529			
Syllable merging 1st item	0.474			
Syllable merging 2nd item	0.662			
Syllable merging 3rd item	0.709			
Syllable merging 4th item	0.762			
Syllable merging 5th item	0.414			
Syllable merging 6th item	0.311			
Initial phoneme identification 1st item				
Initial phoneme identification 2nd item			0.380	
Initial phoneme identification 3rd item			0.351	
Initial phoneme identification 4th item			0.300	
Initial phoneme identification 5th item				
Initial phoneme identification 6th item				
Rhyme recognition 1st item			0.645	
Rhyme recognition 2nd item			0.736	
Rhyme recognition 3rd item			0.561	
Rhyme recognition 4th item			0.589	
Rhyme recognition 5th item			0.480	
Rhyme recognition 6th item			0.469	
Phoneme segmentation 1st item	0.391			
Phoneme segmentation 2nd item				0.478
Phoneme segmentation 3rd item				0.733
Phoneme segmentation 4th item				0.701
Phoneme segmentation 5th item				0.791
Phoneme segmentation 6th item				0.581
Phoneme elimination 1st item		0.512		
Phoneme elimination 2nd item		0.522		
Phoneme elimination 3rd item		0.699		
Phoneme elimination 4th item		0.509		
Phoneme elimination 5th item		0.539		
Phoneme elimination 6th item		0.555		
Phoneme substitution 1st item		0.434		
Phoneme substitution 2nd item		0.570		
Phoneme substitution 3rd item		0.638		
Phoneme substitution 4th item		0.672		
Phoneme substitution 5th item		0.597		
Phoneme substitution 6th item		0.626		

**Table 3:** The reliability of the applied Gray Oral Reading Test GORT-5 scales.

Scales		Alpha
GORT-5 Oral Reading Test	Reading rate	0.70
	Reading accuracy	0.56
	Reading fluency	0.36
Reading comprehension		0.88
Oral Reading Index ORI		0.91

**Table 4:** Reading performance in relation to the class that students attend.

Variable	Grade	N	Mean	SD	F	*p*-Level	*d*
Oral Reading Index (ORI)	1	244	91.55	11.83	0.45	0.64	0.001
	2	210	90.80	12.26			
	3	235	91.86	12.13			
	Total	689	91.43	12.06			

SD-standard deviation; F-Multivariate analysis of variance; Cohen’s d- the effect size measure.

**Table 5:** Phonological awareness based on the grade level.

		Phonological Awareness Categories					Overall	χ2 = 378.71 *p* < 0.001
		Below Average	Low Average	Average	High Average	Above Average		
1	Frekvencija	16	14	51	37	126	244	
	Std. Residual	0.6	3.8	0.6	−8.4	12.2		
2	Frekvencija	18	1	48	143	0	210	
	Std. Residual	1.8	−1.7	1.2	2.6	−6.2		
3	Frekvencija	5	0	33	197	0	235	
	Std. Residual	−2.3	−2.3	−1.8	6.0	−6.6		
		39	15	132	377	126	689	

**Table 6:** The results on the phonological awareness test subscales by grades.

Factor		Arithmetic Mean	SD	F	df	*p*-Level	Eta^2^
Syllable awareness	1st grade	−0.491	1.245	61.216	2/682	<0.001	0.153
	2nd grade	0.091	0.850				
	3rd grade	0.431	0.482				
Advanced phoneme awareness	1st grade	−0.388	1.206	39.146	2/682	<0.001	0.103
	2nd grade	0.0302	0.957				
	3rd grade	0.3781	0.551				
The initial phoneme awareness	1st grade	−0.276	1.461	15.175	2/682	<0.001	0.043
	2nd grade	0.133	0.682				
	3rd grade	0.169	0.412				
Phoneme segmentation	1st grade	−0.248	1.425	12.558	2/682	<0.001	0.036
	2nd grade	0.090	0.864				
	3rd grade	0.178	0.227				

df-degrees of freedom; Eta2-the effect size measure.

**Table 7:** Differences in the phonological awareness of students with and without reading difficulties.

	Reading Difficulties	Mean	SD	F	*p*-Level	*d*
The FONT total	Exists	40.15	8.26	7.53	<0.001	0.054
	Missing	44.30	6.15			
Syllable awareness	Exists	−0.248	1.146	38.193	<0.001	0.053
	Missing	0.212	0.798			
Advanced phonemic awareness	Exists	−0.128	1.207	9.812	0.002	0.014
	Missing	0.109	0.764			
The initial phoneme awareness	Exists	−0.045	1.217	1.233	0.267	0.002
	Missing	0.039	0.766			
Phoneme segmentation	Exists	−0.043	1.242	1.131	0.288	0.002
	Missing	0.037	0.732			

Cohen’s d, the effect size measure.

**Table 8:** Characteristics of multiple-regression analysis.

Model	R	R^2^	F	*p*
1	0.269	0.07	13.356	<0.001

R—correlation coefficient; R²—coefficient of determination.

**Table 9:** Partial contributions of predictors.

Predictors	Beta (β)	*p*-nivo
Syllable awareness	−0.030	0.738
Advanced phoneme awareness	0.096	0.046
The initial phoneme awareness	0.212	<0.001
Phoneme segmentation	0.002	0.981

Criterion—ORI (Oral Reading Index).

## Data Availability

The data presented in this study are available on request from the corresponding author. The data are not publicly available due to privacy.

## References

[1] Golubović S. (2004). Fonološko procesiranje kod dece sa jezičkim poremećajima i poremećajima čitanja. Pedagogija.

[2] Avdyli R., Cuetos F. (2012). Reading difficulties in Albanian. Ann. Dyslexia.

[3] Hagtvet B.E. (1997). Phonological and Linguistic–Cognitive Precursors of Reading Abilities. Dyslexia.

[4] Müller K., Brady S. (2001). Correlates of early reading performance in a transparent orthography. Read. Writ. Interdiscip. J..

[5] Raman I., Weekes B.S. (2005). Acquired Dyslexia in a Turkish-English Speaker. Ann. Dyslexia.

[6] Seymour P.H.K., Aro M., Erskine J.M. (2003). Foundation literacy acquisition in European orthographies. Br. J. Psychol..

[7] Elbeheri G., Everatt J., Mahfoudhi A., Al-Diyar M.A., Taibah N. (2011). Orthographic Processing and Reading Comprehension Among Arabic Speaking Mainstream and LD Children. Dyslexia.

[8] Subotić L.J., Sredojević D., Bjelaković I. (2012). Fonetika i Fonologija: Ortoepska i Ortografska Norma Standardnog Srpskog Jezika.

[9] Al Mannai H., Everatt J. (2005). Phonological Processing Skills as Predictors of Literacy amongst Arabic Speaking Bahraini Children. Dyslexia.

[10] Catts H.W., Fey M.E., Zhang X., Tomblin J.B. (2001). Estimating the risk of future reading difficulties in kinder-garten children: A research-based model and its clinical implementation. Lang. Speech Hear. Serv. Sch..

[11] Chiat S., Roy P. (2008). Early phonological and sociocognitive skills as predictors of later language and social communication outcomes. J. Child Psychol. Psychiatry.

[12] Ivšac Pavliša J., Lenček M. (2011). Fonološke vještine i fonološko pamćenje: Neke razlike između djece urednoga jezičkoga razvoja, djece s perinatalnim oštećenjem mozga i djece s posebnim jezičkim teškoćama kao temeljni prediktor čitanja. Hrvat. Rev. Rehabil. Istraživanja.

[13] McDowell K.D., Lonigan C.J., Goldstein H. (2007). Relations among socioeconomic status, age, and predictors of phonological awareness. J. Speech Lang. Hear. Res..

[14] Miloshevic N., Vukovic M. (2017). Rapid naming in children with specific language impairment and in children with typical language development. J. Spéc. Educ. Rehabilit..

[15] Snowling M. (2000). Dyslexia.

[16] Torgesen J.K., Alexander A.W., Wagner R.K., Rashotte C.A., Voeller K.K.S., Conway T. (2001). Intensive remedial instruction for children with severe reading disabilities: Immediate and long-term outcomes from two instructional approaches. J. Learn. Disabil..

[17] Castles A., Coltheart M. (2004). Is there a causal link from phonological awareness to success in learning to read?. Cognition.

[18] Kodzopeljić J. (2013). Teorijski i primenjeni aspekti psihologije čitanja. Pretpostavke za Usvajanje Veštine Čitanja.

[19] Subotić S. (2011). Konstrukcija testa fonološke svijesti na srpskom jeziku. Primen. Psihol..

[20] Lalović D. (2008). Jezik i Individualne Razlike.

[21] Manis F.R., Seidenberg M.S., Doi L.M. (1999). Rapid naming and the longitudinal prediction of reading subskills in first and second graders. Sci. Stud. Read..

[22] Wagner R.K., Torgesen J.K., Rashotte C.A. (1994). Development of reading-related phonological processing abilities: New evidence of bidirectional causality from a latent variable longitudinal study. Dev. Psychol..

[23] Catts H.W., Gillispie M., Leonard L.B., Kail R.V., Miller C.A. (2002). The role of speed of pro-cessing, rapid naming, and phonological awareness in reading achievement. J. Learn. Disabil..

[24] Snider V.E. (1995). A primer on phonemic awareness: What it is, why it’s important, and how to teach it. Sch. Psychol. Rev..

[25] Anthony J.L., Lonigan C.J. (2004). The Nature of Phonological Awareness: Converging Evidence from Four Studies of Preschools and Early Grade School Children. J. Educ. Psychol..

[26] Gabrieli J.D.E. (2009). Dyslexia: A new synergy between education and cognitive neuroscience. Science.

[27] Lallier M., Donnadieu S., Valdois S. (2012). Developmental dyslexia: Exploring how much phonological and visual attention span disorders are linked to simultaneous auditory processing deficits. Ann. Dyslexia.

[28] Kuppen S.E.A., Goswami U. (2016). Developmental Trajectories for Children with Dyslexia and Low IQ Poor Readers. Dev. Psychol..

[29] Muter V. (2004). Turner and Rack. The Study of Dyslexia.

[30] Gharaibeh M., Sartawi A.A., Dodeen H., Alzyoudi M. (2021). Effects of rapid automatized naming and phonological awareness deficits on the reading ability of Arabic-speaking elementary students. Appl. Neuropsychol. Child.

[31] Logan J.A.R., Schatschneider C., Wagner R.K. (2011). Rapid serial naming and reading ability: The role of lexical access. Read. Writ..

[32] Snowling M.J., Hulme C. (2012). Children’s reading impairments: From theory to practice. Jpn. Psychol. Res..

[33] Raven J., Raven J.C., Court J.H. (1998). Priručnik za Ravenove Progresivne Matrice i Ljestvice Rječnika.

[34] Wiederholt J.L., Bryant B.R. (2012). Gray Oral Reading Tests.

[35] Hall A.H., Tannebaum R.P. (2013). Test Review: J. L. Wiederholt & B. R. Bryant. (2012). Gray Oral Reading Tests—Fifth Edition (GORT-5). Austin, TX: Pro-Ed. J. Psychoeduc. Ass..

[36] Beaton D.E., Bombardier C., Guillemin F., Ferraz M.B. (2000). Guidelines for the Process of Cross-Cultural Adaptation of Self-Report Measures. Spine.

[37] Defior S., Martos F., Cary L. (2002). Differences in reading acquisition development in two shallow orthographies: Portuguese and Spanish. Appl. Psycholinguist..

[38] Golubović S. (2011). Disleksija, Disgrafija, Dispraksija.

[39] Serrano F., Defior S. (2008). Dyslexia speed problems in a transparent orthography. Ann. Dyslexia.

[40] Wimmer H. (1993). Characteristics of developmental dyslexia in a regular writing system. Appl. Psycholinguist..

[41] Van Den Bos K.P. (1998). IQ, Phonological Awareness and Continuous-naming Speed Related to Dutch Poor Decoding Children’s Performance on Two Word Identification Tests. Dyslexia.

[42] Bryant P. (2002). It doesn’t matter whether onset and rime predicts reading better than phoneme awareness does or vice versa. J. Exp. Child Psychol..

[43] Ho C.S.-H., Leung M.-T., Cheung H. (2011). Early Difficulties of Chinese Preschoolers at Familial Risk for Dyslexia: Deficits in Oral Language, Phonological Processing Skills, and Print related Skills. Dyslexia.

[44] Goswami U., Thomson J., Richardson U., Stainthorp R., Hughes D., Rosen S., Scott S.K. (2002). Amplitude envelope onsets and developmental dyslexia: A new hypothesis. Proc. Natl. Acad. Sci. USA.

[45] Siok W.T., Fletcher P. (2001). The role of phonological awareness and visual-orthographic skills in Chinese reading acquisition. Dev. Psychol..

[46] Cassady J., Smith L., Huber L. (2005). Enhancing validity in phonological awareness assessment through computer–supported testing. Pract. Ass. Res. Eval..

[47] Rathvon N. (2004). Early Reading Assessment: A Practitioner’s Handbook.

[48] Cossu G., Shankweiler D., Liberman I.Y., Katz L., Tola G. (1988). Awareness of phonological segments and reading ability in Italian children. Appl. Psycholinguist..

[49] Saksida A., Iannuzzi S., Bogliotti C., Chaix Y., Démonet J.F., Bricout L., Billard C., Nguyen-Morel M.A., Le Heuzey M.F., Soares-Boucaud I. (2016). Phonological Skills, Visual Attention Span, and Visual Stress in Developmental Dyslexia. Dev. Psychol..

[50] Goswami U. (2002). Phonology, reading development, and dyslexia: A cross-linguistic perspective. Ann. Dyslexia.

[51] Lundberg I., Stanovich K.E., Bjaalid I.-K., Høien T. (1995). Components of phonological awareness. Read. Writ..

[52] Harris M., Giannouli V., Harris M., Hatano G. (1999). Learning to read and spell Greek: The importance of letter knowledge and morphological awareness. Learning to Read and Write: A Cross-Linguistic Perspective.

[53] Dębska A., Łuniewska M., Chyl K., Banaszkiewicz A., Żelechowska A., Wypych M., Marchewka A., Pugh K.R., Jednoróg K. (2016). Neural basis of phonological awareness in beginning readers with familial risk of dyslexia—Results from shallow orthography. NeuroImage.

[54] Le Jan G., Le Bouquin-Jeannès R., Costet N., Trolès N., Scalart P., Pichancourt D., Faucon G., Gombert J.-E. (2010). Multivariate predictive model for dyslexia diagnosis. Ann. Dyslexia.

[55] Afflerbach P. (2016). Reading Assessment: Looking Ahed. Read. Teach..

[56] Layes S., LaLonde R., Rebai M. (2020). Reading-related abilities underlying phonological awareness: A cross-sectional study in children with and without dyslexia. Logop. Phoniatr. Vocol..

[57] Snowling M.J. (2013). Early identification and interventions for dyslexia: A contemporary view. J. Res. Spéc. Educ. Needs.

